# Education programmes on performance-based assessment for allied health and nursing clinical educators: A scoping review protocol

**DOI:** 10.12688/hrbopenres.13669.2

**Published:** 2024-01-12

**Authors:** Lucy Alpine, Emer Barrett, Julie Broderick, David Mockler, Anne O'Connor

**Affiliations:** 1 Discipline of Physiotherapy, Trinity College Dublin, Trinity Center for Health Sciences, Dublin, D08W9RT, Ireland; 2 John Sterne Library, Trinity College Dublin, Trinity Center for Health Sciences, Dublin, D08W9RT, Ireland; 3 Munster Physiotherapy Clinic, Limerick, V94R9VW, Ireland

**Keywords:** performance-based assessment, education/training programmes, clinical educators

## Abstract

**Background:**

Performance-based assessment (PBA) is a complex process undertaken in the workplace by healthcare practitioners known as clinical educators, who assist universities in determining health professional students’ readiness for independent practice. Preparing healthcare professionals for PBA is considered essential to ensuring the quality of the assessment process in the clinical learning environment. A preliminary search of the literature indicated a paucity of research guiding the development of education programmes that support practice educators to understand and implement PBA.

**Objective:**

The aim of this scoping review is to investigate and describe education programmes delivered to allied health and nursing clinical educators, to develop PBA knowledge and skills.

**Methods:**

This review will follow the Joanna Briggs Institute (JBI) methodology for conducting scoping reviews. Electronic databases relevant to this research topic will be searched including, EMBASE, ERIC, MEDLINE (Ovid), Web of Science and CINAHL and other targeted databases for grey literature. Studies that include PBA as the main focus or a component of the education programmes, of any format, delivered to clinical educators in allied health and nursing will be included. Studies may report the design and/or implementation and/or evaluation of PBA education programmes. Relevant English language publications will be sought from January 2000 to October 2022. Two reviewers will screen all titles and abstracts against the inclusion/exclusion criteria, and publications deemed relevant will be eligible for full text screening, confirming appropriateness for inclusion in the scoping review. Data will be charted to create a table of the results, supported a by narrative summary of the findings in line with the review objectives.

## Introduction

Assessment of health professional students during work-based placements, also known as performance-based assessment (PBA), refers to the appraisal process conducted during clinical placements, and establishes students’ readiness for independent professional practice ^1^. PBA determines health professional competence ^2^, providing evidence of students’ abilities to care for patients in a real, uncontrolled clinical learning environment ^3^. This assessment process aligns with effective outcome-based education, where students are required to achieve standardised professional competencies in order to graduate as competent clinicians ^2^. Preliminary searches of relevant literature highlighted a paucity of evidence-based education programmes for clinical educators. Clinical educators are healthcare practitioners primarily involved in service delivery, who undertake student supervision and PBA in the workplace ^4^. Titles used to describe health professionals who undertake this education role include practice educator, preceptor, mentor, fieldwork educator, clinical teacher and clinical educator ^5– 8^. For consistency, the term ‘clinical educator’ will be used throughout this protocol.

Current literature in this area highlights challenges with PBA, citing inter and intra-rater reliability and knowledge of assessment processes as the greatest difficulties ^9– 12^. Clinical educators who are deficient in knowledge, skills and confidence for this role, may potentially compromise the PBA decision-making process, thereby casting doubt on a graduate’s credentials as a safe competent practitioner. Thus, exploration of current opportunities available for health professionals to address their educational needs in this area is most important ^6, 13, 14^.

Assessment in the clinical environment is a complex process open to many challenges and influenced by several factors including the assessor, the clinical learning environment and the assessment task ^9, 11, 15^. Considerable responsibility is placed on these clinicians, as PBA often determines progression through the programme and may contribute significantly to the overall grade awarded to the student ^16, 17^. Dynamics impacting PBA relating to the assessors may include, variation in interpretation of the task and how it is judged ^18^, differences in understanding of the assessment criteria ^19^, and navigating the complex student-clinician relationship, where assessors act as both mentor and examiner ^9, 18^. Other relevant influences may include potential lack of commitment to the assessment process by clinical educators due to it been seen as a non-service related activity ^20^, and cognitive or cultural biases ^11, 18^. In the clinical learning environment, issues such as time pressures ^21^, the multiple demanding roles of the clinician ^1^ and the uncertain nature of the clinical setting, often presenting complex learning opportunities ^11^, also present challenges. Assessment tasks may be viewed as problematic, requiring assessors to convert an observed performance into a numerical score ^1, 22^, with the added challenge of precise recall of a performance, due to the time lapse between observation and actual grading ^22^. Furthermore, these difficulties may be heightened when a clinical educator is supervising and grading an underperforming student ^23^. Thus, professional development to support clinical educators to carry out their role as assessors is critical to ensure the quality of work-based education and assessment ^6, 9, 16^.

There is some evidence to suggest that universities provide education programmes to induct new practice educators into their role, and more advanced programmes for experienced educators, to grow their teaching and assessment skills ^7, 14, 15, 24, 25^. Programmes are delivered mainly through formal instructional approaches such as university-based workshops or online e-learning units, which may include education on implementing PBA ^15, 24– 26^. However, certification requirements for these programmes are inconsistent and no specific guidance in relation to the format, content or frequency of PBA education is recommended ^10, 27, 28^.

The majority of research related to PBA education for healthcare professional is situated in medical education ^21, 29, 30^, with preliminary searches suggesting limited studies for allied health professions and nursing ^25, 31– 33^. In medicine, the literature on PBA spans both pre-qualification and post qualification programmes, where the implementation of PBA often differs from that applied in allied heath/nursing education. During medical rotations, students are generally under the supervision of clinical educators for short periods of time ^34, 35^, and commonly use assessment tools such as the Mini-Clinical Examination Exercise (Mini-CEX) and Direct Observation of Procedural Skills (DOPS), applying only a discrete number of assessment criteria ^3, 4, 36, 37^. Clinical performance is evaluated at distinct pre-arranged timepoints, often planned or identified by the student, and verbal and/or written feedback is not always included ^38, 39^. These assessment approaches and tools may not provide an integrated view of overall patient management by the student or reflect care provided over time ^35, 36^. 

In nursing and allied health education, PBA is generally applied as a continuous assessment process over a prolonged period of time, for example 6 to 12 weeks ^40, 41^. Students are often supervised by one/two educators for the duration of the placement and work closely together under their supervision ^40^. PBA generally consists of multiple daily observations of the student, which can include reviewing student’s assessments and clinical reasoning documentation, together with observation of their professional and communication skills ^42, 43^. PBA tools are frequently used for both formative and summative feedback, with written feedback informing the dialogue regarding the students’ performance ^44^. Differences in supervision and assessment practices may suggest educational needs for clinical educators are also different, depending on how the PBA assessment is applied and their clinical learning environment.

Despite the difference in how PBA is applied, a preliminary search of the research suggests similar approaches are used. In medicine, studies frequently use interactive workshops and video vignette of students’ clinical performances based on teaching approaches such as ‘Performance Dimension Training’ or ‘Frame of Reference’ training ^21, 29, 30^. These studies have shown a positive impact on participants assessment confidence, skills and knowledge, with more variable success for grading accuracy and reliability. A small number of recent allied health studies using similar approaches, highlighted the benefits of interactive dialogue between assessors as part of the decision-making process, resulting in more transparent assessment processes, and also improved rating accuracy of assessors ^15, 45^. A preliminary search identified one systematic review specifically associated with professional development of clinical educators. Milne *et al*., (2011) reviewed evidence-based training for clinical supervisors in mental health professions, identifying 20 different topics provided in education programmes, however assessment was not explicitly reported as a component of training in any of these studies ^25^. A further systematic review addressing the issues and concerns related to learning and assessment in the clinical learning environment ^14^, and a more recent scoping review exploring the factors needed to support clinical educators in the workplace ^46^, do not specifically addresses PBA education, however both highlight the requirement for professional development in assessment practices. A search for existing scoping and systematic review protocols related to education programmes on performance-based assessment for allied health or nursing professionals was conducted in MEDLINE (Ovid), Best Evidence Medical Education, Joanna Briggs Institute (JBI) Evidence Synthesis and Cochrane Database of Systematic Reviews in July 2022, producing no protocols on this topic.

Preparing clinical educators to implement PBA and develop expertise as an assessor requires on-going education and experience ^46, 47^. In addition, there is mounting pressure on universities from accreditation and regulatory bodies to ensure clinical educators are fit for their role, which includes the provision of credible and defensible PBA evaluations to safeguard the public. Therefore, it is a timely and essential task to investigate the breath and scope of existing literature regarding the education provided to clinical educators to deliver PBA. The aim of this scoping review is to investigate and describe education programmes delivered to allied health and nursing clinical educators, to develop PBA knowledge and skills. 

## Methods

This proposed scoping review will be conducted in accordance with the Joanna Briggs Institute scoping review guidelines ^48^, informed by the Arskey and O’Malley framework ^49^, with consideration of the recommendations from Levac *et al*., ^50^, Duadt *et al*., ^51^ and Peters *et al*., 2022 ^52^.

### Rationale for scoping review

Scoping reviews are an effective method to establish the extent of the available research on a particular topic by exploring diverse international research sources, clarifying key concepts and identifying gaps in the knowledge on a specific research topic ^51, 53^. In addition, given the limited yield of literature from the initial search and investigative nature of this research, a scoping review is well positioned to identify any gaps in the research.

### Research question, objectives and purpose

This study protocol will address the following question: what is known about education programmes used to develop PBA knowledge and skills for clinical educators in allied health and nursing?

The review objectives are as follows:

- To explore the extent, range and nature of the literature on PBA education programmes provided to allied health and nursing professionals
- To chart specific characteristics related to the context, target population, format, aim, instructional methods/teaching strategies, content, pedagogical approaches, evaluation and key findings or educational impact of these programmes
- To collate, describe and summarise the available evidence on PBA education programmes, and identify any gaps in the existing research

The purpose of the review is to explore and describe education programmes delivered to allied health and nursing clinical educators, to develop PBA knowledge and skills. 

### Inclusion criteria

The search strategy was constructed using the Population-Concept-Context framework to guide the eligibility criteria. An overview of the inclusion and exclusion criteria are outlined in Table 1.

**Table 1.** Inclusion and exclusion criteria.

Inclusion criteria	Exclusion criteria
• Studies that report the design and/or implementation and/or evaluation of PBA education programmes for allied health and nursing clinical educators supervising pre-qualification dietetic, occupational therapy, nursing, optometry, orthoptic, physiotherapy, podiatry, psychology, radiography, radiation therapy, social workers and speech and language therapy students • Qualitative, quantitative, primary or mixed methods research studies of any design • Opinion pieces, reviews, conference abstracts that comment on PBA empirical research or have PBA education as the main focus • Grey literature retrieved from Google Scholar, ResearchGate, GreyLit and Open Grey • Full text papers or reports in the English language • Published after 1 ^st^ January 2000 to the 30 ^th^ of October 2022	• Professional development programmes targeting university academic staff or allied health professionals other than dietitians, occupational therapists, nursing, optometrists, orthoptists, physiotherapists, podiatrists, psychologists, radiographers, radiation therapists, social workers and speech and language therapists • Professional development programmes relating to clinical education that do not include a PBA component • General information regarding PBA on websites of individual allied health or nursing professional bodies, professional regulators, government education departments • Research papers or grey literature not written in English

### Participants

The primary population of this review are clinical educators, described as clinicians who supervise pre-qualification students on clinical placements. Clinical educators may work in a part-time or full-time capacity and be employed across a wide range of workplaces providing healthcare to service users. This scoping review will consider studies that include clinical educators from the following health and social work professions: dietitians, occupational therapists, nursing, optometrists, orthoptists, physiotherapists, podiatrists, psychologists, radiographers, radiation therapists, social workers and speech and language therapists. Professions identified include those awarded a recognised professional entry-level qualification from universities and have work-based placements as an integral part of their programmes.

### Concept

This scoping review is designed to explore PBA education programmes provided to allied health and nursing clinical educators who supervise pre-qualification students. Medical education programmes were not included as their assessment practice in clinical education differ considerably from allied health and nursing. These programmes may be designed, developed and delivered by academics with a practice education remit or dedicated discipline specific practice education staff. Education programmes must include PBA as the main focus or as a component of a programme, giving a clear description of the design and/or implementation and/or evaluation of the assessment aspect. All instructional methods or teaching strategies for programme delivery will be considered and may include but are not limited to, interactive workshops, didactic lectures, structured reflection opportunities, role-play or other simulation approaches, workplace observational coaching or other methods. Evaluation outcomes for the programmes will be pursued.

### Context

PBA education programmes may be based in the university as a face-to-face event, or as a university e-learning virtual programme or facilitated in a clinical setting. Programmes may also vary in duration from single interventions completed over a number of hours or a single day, to more longitudinal programmes delivered over a defined period of time. Literature will be searched from the year 2000 recognising the growth in research and development in the area of work-based assessment in recent decades. There will be no limits on geographical location.

### Search strategy

A professional librarian will be consulted to assist with developing comprehensive search strategies to identify relevant literature from a broad range of sources including electronic databases, reference lists and grey literature. Initial searches of EMBASE and PUBMED databases were undertaken in July 2022 to identify studies relevant to the research area. Keywords and index terms of the relevant studies were then used to create search strategies. The final EMBASE search strategy is outlined in Table 2.

**Table 2.** Search strategy for EMBASE.

Search	Query
S1	(('performance based' OR 'workplace based' OR 'competency based' OR 'performance based') NEAR/3 assess*):ti,ab,kw
S2	('domains of competence' OR 'Competency-Based Education' OR 'Competency-Based assessment*' OR 'competency- based portfolio*' OR ‘assessment of competence’ OR 'frame of reference training' OR 'performance dimension training' OR 'behaviour observation training'):ti,ab,kw
S3	#1 OR #2
S4	'allied health student'/exp OR 'allied health education'/exp
S5	('allied health' NEAR/3 (educat* OR curricul* OR train* OR ‘clinical skill*’ OR competenc* OR evaluat* OR assess*)):ti,ab,kw
S6	('audiology'/exp AND ('clinical competence'/exp AND 'curriculum'/exp AND 'education'/exp)) OR 'audiology student'/exp
S7	(audiol* NEAR/3 (educat* OR curricul* OR train* OR ‘clinical skill*’ OR competenc* OR evaluat* OR assess*)):ti,ab,kw
S8	('dietetics'/exp AND ('clinical competence'/exp AND 'curriculum'/exp AND 'education'/exp)) OR 'dietetics education'/exp OR 'dietetics student'/exp
S9	((dietitian* OR dietetic*) NEAR/3 (educat* OR curricul* OR train* OR ‘clinical skill*’ OR competenc* OR evaluat* OR assess*)): ti,ab,kw
S10	'nursing'/exp OR 'nursing student'/exp OR 'nursing education'/exp OR 'nursing practice'/exp
S11	(Nurs* NEAR/3 (educat* OR curricul* OR train* OR ‘clinical skill*’ OR competenc* OR evaluat* OR assess*)):ti,ab,kw
S12	'occupational therapy'/exp OR 'occupational therapy student'/exp OR 'occupational therapy education'/exp OR 'occupational therapy practice'/exp
S13	('occupational therap*' NEAR/3 (educat* OR curricul* OR train* OR ‘clinical skill*’ OR competenc* OR evaluat* OR assess*)): ti,ab,kw
S14	(('optometry'/exp OR 'orthoptics'/exp) AND ('student'/exp OR 'education'/exp OR 'learning'/exp OR 'clinical education'/exp)) OR 'optometry education'/exp
S15	((Optometr* OR orthoptics) NEAR/3 (educat* OR curricul* OR train* OR ‘clinical skill*’ OR competenc* OR evaluat* OR assess*)):ti,ab,kw
S16	'physiotherapy'/exp OR 'physical therapy student'/exp OR 'physical therapy education'/exp OR 'physiotherapy practice'/exp
S17	((‘physical therap’ OR physiotherap*) NEAR/3 (educat* OR curricul* OR train* OR ‘clinical skill*’ OR competenc* OR evaluat* OR assess*)):ti,ab,kw
S18	('podiatry'/exp AND ('student'/exp OR 'education'/exp OR 'learning'/exp OR 'clinical education'/exp)) OR 'podiatry education'/exp
S19	(podiatr* NEAR/3 (educat* OR curricul* OR train* OR ‘clinical skill*’ OR competenc* OR evaluat* OR assess*)):ti,ab,kw
S20	(('psychology'/exp OR 'psychologist'/exp) AND ('student'/exp OR 'education'/exp OR 'learning'/exp OR 'clinical education'/exp)) OR 'psychology student'/exp OR 'psychology education'/exp
S21	(Psycholog* NEAR/3 (educat* OR curricul* OR train* OR ‘clinical skill*’ OR competenc* OR evaluat* OR assess*)):ti,ab,kw
S22	'radiotherapy'/exp AND ('student'/exp OR 'education'/exp OR 'learning'/exp OR 'clinical education'/exp)
S23	((Radiotherap* OR 'radation therap*') NEAR/3 (educat* OR curricul* OR train* OR ‘clinical skill*’ OR competenc* OR evaluat* OR assess*)):ti,ab,kw
S24	'Radiography'/exp AND ('student'/exp OR 'education'/exp OR 'learning'/exp OR 'clinical education'/exp)
S25	(Radiograph* NEAR/3 (educat* OR curricul* OR train* OR ‘clinical skill*’ OR competenc* OR evaluat* OR assess*)):ti,ab,kw
S26	(('social work'/exp OR 'social worker'/exp OR 'social work practice'/exp) AND ('student'/exp OR 'education'/exp OR 'learning'/exp OR 'clinical education'/exp)) OR 'social work student'/exp
S27	(('social work' OR 'social care work') NEAR/3 (educat* OR curricul* OR train* OR ‘clinical skill*’ OR competenc* OR evaluat* OR assess*)):ti,ab,kw
S28	('speech language pathologist'/exp OR 'speech and language rehabilitation'/exp) AND ('student'/exp OR 'education'/exp OR 'learning'/exp OR 'clinical education'/exp)
S29	(('speech language' OR 'speech and language' OR 'speech & language') NEAR/3 (educat* OR curricul* OR train* OR ‘clinical skill*’ OR competenc* OR evaluat* OR assess*)):ti,ab,kw
S30	#4 OR #5 OR #6 OR #7 OR #8 OR #9 OR #10 OR #11 OR #12 OR #13 OR #14 OR #15 OR #16 OR #17 OR #18 OR #19 OR #20 OR #21 OR #22 OR #23 OR #24 OR #25 OR #26 OR #27 OR #28 OR #29
S31	'program development'/exp OR 'curriculum development'/exp OR 'course content'/exp OR 'education program'/exp OR 'tertiary education'/exp OR 'vocational education'/exp OR 'allied health education'/exp OR 'occupational therapy education'/exp OR 'physical therapy education'/exp OR 'teacher training'/exp OR 'program evaluation'/de
S32	((Program OR curricul* OR pilot*) NEAR/3 (develop* OR evaluat* OR design* OR create* OR initiate*)):ti,ab,kw
S33	((‘allied health’ OR ‘occupational therap*’ OR ‘physical therap’ OR physiotherap* OR dietitian* OR dietetic* OR ‘speech and language’) NEAR/3 (educat* OR curricul* OR train* OR ‘clinical skill*’ OR competenc* OR evaluat* OR assess*)):ti,ab,kw
S34	((‘allied health’ OR ‘occupational therap*’ OR ‘physical therap*’ OR physiotherap* OR dietitian* OR dietetic* OR ‘speech and language’) NEAR/3 (educat* OR curricul* OR train* OR ‘clinical skill*’ OR competenc* OR evaluat* OR assess*)):ti,ab,kw
S35	‘Train the trainer*’:ti,ab,kw
S36	((Student OR undergraduate* OR postgraduate*) NEAR/3 (educat* OR curricul* OR train* OR ‘clinical skill*’ OR competenc* OR evaluat* OR assess*)):ti,ab,kw
S37	((Teacher* OR instructor* OR clinician* OR mentor* OR faculty OR educator*) NEAR/3 (train* OR develop* OR skill* OR tool*)):ti,ab,kw
S38	#31 OR #32 OR #33 OR #34 OR #35 OR #36 OR #37
S39	#3 AND #30 AND #38

A comprehensive search will be undertaken to identify relevant publications in the following databases: EMBASE, ERIC, MEDLINE (Ovid), Web of Science and CINAHL. Bibliographies of relevant publications and review articles will be examined for further citations and checked for suitability using the eligibility criteria. A grey literature search will be undertaken using databases identified in the inclusion criteria. Websites of individual professional bodies, universities, professional regulator or government education departments will not be searched as this is beyond the resources of this research project, considering the breath of professions included in the review. The search will be iterative and any relevant additional terms, concepts or literature sources, emerging during the search, screening or selection processes, may be added to the search strategy as the review progresses ^52^. Only publications in the English language will be included.

### Sources of evidence selection

Following the searches, appropriate publications will be imported into Covidence, and any duplicates not already detected will be removed ( Abstrackr is a free open source alternative that can carry out similar functions to Covidence). To ensure reliability in selection, two reviewers will independently screen the title and abstract of a random sample of 20 retrieved articles, to ensure consistency between the reviewers regarding the eligibility criteria ^52^. All records will then be screened by two reviewers, with titles and abstracts appraised against the inclusion/exclusion criteria ^51^. Uncertainties or disputes related to the study selection will be discussed at scheduled meetings at the beginning, midpoint and final stages of the abstract review process ^50^. Where an inclusion or exclusion decision cannot be agreed from the title or abstract, the full article will be screened. Publications deemed appropriate will be eligible for full text screening, by the two independent reviewers, confirming appropriateness for inclusion in the scoping review. Any disagreements will be resolved through discussion and consensus with a third reviewer.

### Data extraction

Covidence will be used to manage citations and perform data extraction based on pre-determined data characteristics listed below. Initially, five to ten identified papers will be piloted by two reviewers to ensure agreement on data extraction characteristics and that the data fields are consistent with the research question ^50^. During the review process, data fields may be further refined with any changes reported in the final review ^49, 54^. Any questions will be resolved by discussion to reach consensus. Where ambiguity or omissions exist, study authors will be contacted for further details. Data will be extracted by a single review author and verified by a second reviewer. As the purpose of this scoping review is focused on describing sources of evidence on a specific research topic, a risk of bias for source of evidence will not be included ^49, 52^.

The following fields will be extracted from the included studies:

**1.**  Author
**2.**  Year of publication
**3.**  Country of study
**4.**  Healthcare profession
**5.**  Size of study population
**6.**  Format/type of programme
**7.**  Location and duration of progamme
**8.**  Overall aim or focus of the programme
**9.**  Instructional methods/teaching strategies
**10.**  Content of programme
**11.**  Theoretical/conceptual framework/pedagogies underpinning teaching methods
**12.**  Evaluation methods
**13.**  Key findings/educational impact

### Data analysis and presentations

The Preferred Reporting Items for Systematic Reviews and Meta-Analyses extension for Scoping Review (PRIMSA-ScR) checklist will be followed for organisation of information and reporting ^55^. A PRISMA flow diagram will be used to report the results of the literature search. This will include numbers of articles imported, duplicates identified, titles and abstracts screened, irrelevant studies removed, rationale for exclusion of articles and final full-text studies included for review ^56^. To maintain transparency, any changes from the protocol will be reported in the scoping review report ^52^. All data extracted will be mapped into tabular format, based on descriptive headings in the data extraction form, and accompanied by a narrative summary of the results in line with the review objectives. A comparison of allied health/nursing and medicine PBA education programmes will be included in the discussion.

### Study status

At the time of publication of this review protocol, title and abstract screening were underway.

## Data Availability

No data are associated with this article.
