# Supplementary material for: Education programmes on performance-based assessment for allied health and nursing clinical educators: A scoping review protocol
Source: HRB Open Res. 2024 Jan 12;6:11. Originally published 2023 Feb 7. [Version 2] doi: 10.12688/hrbopenres.13669.2 (PMC11791400; doi:10.12688/hrbopenres.13669.2)
Supplement: Peer review history

# Reviewer report: Reviewer response for version 2

**DOI:** 10.21956/hrbopenres.15187.r44454

**Published:** 2025-02-03

**Authors:** Susanne Friis Søndergaard

**Affiliations:** 1 Viborg Regional Hospital, Aarhus University, Roskilde University, Roskilde, Denmark

Thank you for the opportunity to review your protocol. I find this a very interesting topic and believe it is important to provide an overview of the existing literature.

 This Scoping Review (ScR) protocol describes a study aiming to investigate and describe education programs delivered to allied health and nursing clinical educators to develop PBA knowledge and skills. The authors tend to follow the Joanna Briggs guidelines for conducting a ScR.

 The aim of the study is clearly stated; however, the rationale for conducting this ScR seems rather unclear. I suggest that the authors revise the background section to make it more transparent. Consider adding subheadings to clarify why the aim and the research questions are stated as they are. It would be appropriate to establish a clearer connection between the background and the research questions.

 I also noticed a lack of information on how you will address non-scientific and grey literature. It might be appropriate to include a section on the extraction of these types of sources. Additionally, please address the issue that the Open grey database is no longer active and how you plan to handle this.

 Furthermore, I suggest providing a clearer statement of the expected contributions of each author to different parts of the study.

 Overall, I congratulate you on a well-done job.

Is the study design appropriate for the research question?

Yes

Is the rationale for, and objectives of, the study clearly described?

Yes

Are sufficient details of the methods provided to allow replication by others?

Yes

Are the datasets clearly presented in a useable and accessible format?

Not applicable

Reviewer Expertise:

Nursing, Education, Hospital design, Advanced nursing practice, Perioperative nursing

I confirm that I have read this submission and believe that I have an appropriate level of expertise to confirm that it is of an acceptable scientific standard, however I have significant reservations, as outlined above.

# Reviewer report: Reviewer response for version 2

**DOI:** 10.21956/hrbopenres.15187.r37759

**Published:** 2024-01-30

**Authors:** Lindy L. McAllister

**Affiliations:** 1 Faculty of Medicine and Health, University of Sydney, Sydney, NSW, Australia

The authors have satisfactorily revised the paper to explain why they excluded literature from medicine in their review, and corrected typos.

Is the study design appropriate for the research question?

Yes

Is the rationale for, and objectives of, the study clearly described?

Yes

Are sufficient details of the methods provided to allow replication by others?

Yes

Are the datasets clearly presented in a useable and accessible format?

Yes

Reviewer Expertise:

NA

I confirm that I have read this submission and believe that I have an appropriate level of expertise to confirm that it is of an acceptable scientific standard.

# Reviewer report: Reviewer response for version 1

**DOI:** 10.21956/hrbopenres.14948.r36233

**Published:** 2023-10-18

**Authors:** Yvonne Finn

**Affiliations:** 1 University of Galway, Galway, Ireland

Rationale: The rationale for this scoping review is presented well. The multifaceted challenges presented to clinical educators in assessing BPA are clearly outlined. The need for educational training in PBA for these clinical assessors is made.

 Can the authors explain how they view the evidence from BPA education in medical education and/or how, if at all, this related to BPA education in nursing and allied health?

 Research question: The research question is clearly stated.

 Methods: The study design and methods are described well.

 Can the authors consider the relevance of reporting key findings/impact of identified PBA education programmes as one of the objectives? This may be challenging to interpret in view of the types of studies listed in the inclusion criteria and the authors’ intention not to do a critical appraisal of included studies. A focus on the impact of interventions is not usually included in scoping reviews for these reasons. At the same time, it is appropriate to report/summarise the key findings as they relate to the scoping review research question.

 Please review the purpose of reference to Abstrackr. Do the authors intend to use this?

 Please not the Preferred Reporting Items for Systematic Reviews and Meta-Analyses extension for Scoping Review checklist is abbreviated to PRISMA-ScR.

Is the study design appropriate for the research question?

Yes

Is the rationale for, and objectives of, the study clearly described?

Partly

Are sufficient details of the methods provided to allow replication by others?

Yes

Are the datasets clearly presented in a useable and accessible format?

Not applicable

Reviewer Expertise:

NA

I confirm that I have read this submission and believe that I have an appropriate level of expertise to confirm that it is of an acceptable scientific standard, however I have significant reservations, as outlined above.

# Response

**Published:** 2024-01-09

**Authors:** Lucy Alpine

**Affiliations:** Trinity College Dublin, Ireland

**Response to Reviewer 2: Dr Joanne Finn**

**Review comment 8:** Rationale - The rationale for this scoping review is presented well. The multifaceted challenges presented to clinical educators in assessing BPA are clearly outlined. The need for educational training in PBA for these clinical assessors is made.

- **Response:** Thank you for taking time to review this protocol and for your valuable comments.

**Review Comment 9: **Can the authors explain how they view the evidence from BPA education in medical education and/ **or** how, if at all, this related to BPA education in nursing and allied health?

- **Response:** Please see our response to reviewer 1, comment 2.  As outlined, we have included further details in the Introduction section of the protocol.

**Review comment 10:** Research question - The research question is clearly stated.

- **Response: **Thank you for your helpful comment.

**Review comment 1**1: Methods: The study design and methods are described well.

- **Response: **Thank you for your supportive comment.

**Review comment 12:** Can the authors consider the relevance of reporting key findings/impact of identified PBA education programmes as one of the objectives?

- **Response:** Thank you for your comment. The key findings/impact of PBA education programmes are not included as a specific objective in line with the definitions and purposes of a scoping review (Levac et al, 2010, Peters et al, 2022). The broad objective of this scoping review is to investigate and describe the range and characteristics of PBA programmes, identifying commonalities and differences. The review will include studies of all designs, including grey literature and without a specific focus on RCTs, the effectiveness of PBA programmes cannot therefore be specifically evaluated. However, key findings and educational impact have been included in the data extraction fields and may assist with the objectives of recognising gaps in the existing research and determining the potential value of a full systematic review on this topic (Levac et al, 2010).

**Review comment 13:** Please review the purpose of reference to Abstrackr. Do the authors intend to use this?

- **Response:** Please see our response to reviewer 1, comment 4. 

**Review comments 14:** Please not the Preferred Reporting Items for Systematic Reviews and Meta-Analyses extension for Scoping Review checklist is abbreviated to PRISMA-ScR.

- **Response:** Thank you for your comment. We have corrected the text.

# Reviewer report: Reviewer response for version 1

**DOI:** 10.21956/hrbopenres.14948.r35621

**Published:** 2023-09-08

**Authors:** Lindy L. McAllister

**Affiliations:** 1 Faculty of Medicine and Health, University of Sydney, Sydney, NSW, Australia

A clearly written protocol to add to knowledge on an important topic.

 It is not clear why the decision was taken to exclude papers from medicine, which as authors says has addressed this topic. This would allow a compare and contrast of findings from an area with little literature - nursing and allied health professions, to an area with more literature – medical education. Or will this compare and contrast be done in Discussion section of subsequent paper which presents the scoping review.

 Comprehensive literature introduces the gap in research on education of assessors to use PBA.

 Good argument made for a scoping review rather than systematic review.

 Search strategy is comprehensive and logical and search terms should pick up all possible papers in the specific nursing and allied health professions.

 Not sure why the sentence on Abstracker is included given it does not appear this software will be used.

 There are a few typos eg guidance …. Are instead of guidance …. Is; missing plural s on recommendation.

Is the study design appropriate for the research question?

Yes

Is the rationale for, and objectives of, the study clearly described?

Yes

Are sufficient details of the methods provided to allow replication by others?

Yes

Are the datasets clearly presented in a useable and accessible format?

Yes

Reviewer Expertise:

NA

I confirm that I have read this submission and believe that I have an appropriate level of expertise to confirm that it is of an acceptable scientific standard, however I have significant reservations, as outlined above.

# Response

**Published:** 2024-01-09

**Authors:** Lucy Alpine

**Affiliations:** Trinity College Dublin, Ireland

**Response to Reviewer 1: Prof. Lindy McAllister**

**Review comment 1:** A clearly written protocol to add to knowledge on an important topic.

- **Response: **Thank you for taking time to review this protocol and for your valuable comments.

**Review comment 2:** It is not clear why the decision was taken to exclude papers from medicine, which as authors says has addressed this topic. This would allow a compare and contrast of findings from an area with little literature - nursing and allied health professions, to an area with more literature – medical education. Or will this compare and contrast be done in Discussion section of subsequent paper which presents the scoping review.

- **Response: **Thank you for your comments. The research team debated the decision to include medical education at length prior to commencing the study. In the end, we decided that the differences in the assessment and supervision processes of medical and allied health/nursing students may have different implications for the content of education programmes addressing PBA and thus the scoping review.

A decision was therefore taken to limit the scoping review to allied health/nursing professions only, with a view to providing a clear review of the literature of these professions. We agree, we could have provided clearer justification for this in the introduction and have added further details to this section.

 We have also now clearly articulated our intention to compare our scoping review findings with that of the medical literature in the Data Analysis and Presentations section.

**Review comment 3:** Comprehensive literature introduces the gap in research on education of assessors to use PBA.

- **Response: **Thank you for your supportive comment.

**Review comment 4:** Good argument made for a scoping review rather than systematic review.

- **Response: **Thank you for your supportive comment.

**Review comment 5:** Search strategy is comprehensive and logical and search terms should pick up all possible papers in the specific nursing and allied health professions.

- **Response: **Thank you for your helpful comment.

**Review comment 6**: Not sure why the sentence on Abstracker is included given it does not appear this software will be used.

- **Response: **The authors understood that a free online data collection and organisation tool should be identified in the protocol. The Editorial Team of HRB Open have clarified they have an open data policy and require all supporting data to made available on publication. This will be achieved through depositing our publication in the TCD Tara Institutional Repository. The reference to Abstracker has therefore been removed from the protocol.

**Review comment 7:** There are a few typos e.g., guidance …. Are instead of guidance …. Is; missing plural s on recommendation.

- **Response: **Thank you, we have proofread the article and corrected the typos.
